# The Impact of Blood Sample Processing on Ribonucleic Acid (RNA) Sequencing

**DOI:** 10.3390/genes15040502

**Published:** 2024-04-17

**Authors:** Zhiyu Liu, Tinglan Ouyang, Yuwei Yang, Yuqi Sheng, Huajuan Shi, Quanjun Liu, Yunfei Bai, Qinyu Ge

**Affiliations:** State Key Laboratory of Digital Medical Engineering, Southeast University, Nanjing 211189, China; 15195986641@163.com (Z.L.); 220171818@seu.edu.cn (T.O.); yyw.roy.w@gmail.com (Y.Y.); sheng_yuqi@163.com (Y.S.); 18705168526@163.com (H.S.); lqj@seu.edu.cn (Q.L.); whitecf@seu.edu.cn (Y.B.)

**Keywords:** blood sample processing, RNA-Seq, sample heterogeneity, time-sensitive genes

## Abstract

In gene quantification and expression analysis, issues with sample selection and processing can be serious, as they can easily introduce irrelevant variables and lead to ambiguous results. This study aims to investigate the extent and mechanism of the impact of sample selection and processing on ribonucleic acid (RNA) sequencing. RNA from PBMCs and blood samples was investigated in this study. The integrity of this RNA was measured under different storage times. All the samples underwent high-throughput sequencing for comprehensive evaluation. The differentially expressed genes and their potential functions were analyzed after the samples were placed at room temperature for 0h, 4h and 8h, and different feature changes in these samples were also revealed. The sequencing results showed that the differences in gene expression were higher with an increased storage time, while the total number of genes detected did not change significantly. There were five genes showing gradient patterns over different storage times, all of which were protein-coding genes that had not been mentioned in previous studies. The effect of different storage times on seemingly the same samples was analyzed in this present study. This research, therefore, provides a theoretical basis for the long-term consideration of whether sample processing should be adequately addressed.

## 1. Introduction

At present, there is plenty of evidence that RNA-sequencing (RNA-Seq) results can be used to assist in early cancer diagnosis, to provide references for precision medication and to reduce the occurrence of adverse drug reactions; these are benefits of the high level of accuracy of next-generation sequencing, which can reach 99.99% [1,2,3,4,5]. At the same time, the continuously decreasing cost of RNA-Seq lowers the barrier of application of sequencing data. Furthermore, many stable and accurate software applications and packages for the bioinformatic analysis of RNA-Seq data have emerged since the first RNA-Seq was performed in 2006 [6,7,8,9,10], opening the way for a period of rapid development. However, there is still no gold standard on how the samples for RNA-Seq are processed according to different needs.

The factors that affect the quality of RNA sequencing data include RNA extraction and storage, from samples to subsequent sequencing [10]. Generally speaking, obtaining RNA-sequencing data from samples requires a process of sample acquisition, transportation, storage, RNA extraction, library construction, sequencing and analysis. During these sample preparation procedures, new neglected variables may be introduced, which can then affect the precision of the RNA-Seq results [11,12,13]. Researchers have found that PBMCs stored for different amounts of time have different transcriptome results, which hinder the identification and interpretation of cancer-specific alterations [14]. The tumor and development-process marker genes *LEF1* and *PHF20* have a tendency to change depending on storage time, which will affect the accuracy of correct tumor-marker screening or early screening. Dvinge and colleagues [14] pointed out that incubating samples on ice significantly reduced the rate of transcriptome changes over time. After being placed on ice for 48 h, the amplitude of differential gene expression and alternative splicing was approximately equivalent to the change after 4 h at room temperature. Pogosova et al. concluded that cryopreserved acute myeloid leukemia (AML) samples had extensive heterogeneity in terms of the survival rate, the percentage of non-leukemia cells and the overall differentiation status of leukemia cells [15]. Fowles and colleagues found that elderly and young patients with polycythemia vera had different genomic characteristics. The frequency of *JAK2 V617F* allele mutations in the elderly population was significantly higher than that in the younger population. This also shows that, when using different individuals for the control group and experimental group is unavoidable, we should try to maintain the consistency of gender, age and other characteristics [16].

In general, research on the influencing factors of RNA sequencing quality has not been elaborated yet. There are many problems waiting to be optimized and discussed. Among them, one often-overlooked aspect is the sample preparation process, which may bring potential variations and deviations into RNA-Seq experiments.

The commonly used technique for the extraction of RNA from blood requires the separation of PBMCs. This process increases the complexity of the entire extraction. The more steps there are in such a process, the easier it is to accumulate errors and the more time-consuming labor there is. At this stage, mature commercial kits for separating PBMCs from blood have not yet been developed. The commonly used method is to remove the white membrane layer after centrifugation, which results in low reproducibility and large differences in operations between different experimenters. This has little effect on qualitative experiments but has an impact on quantitative experiments. Because additional steps will introduce new variables, the accuracy of quantitative experiments, such as RNA-Seq, will be affected by the added variables.

The reagent kit for extracting whole blood is relatively thorough. Although it is more convenient and faster to extract RNA directly from the blood instead of isolating PBMCs, different kits may affect the quality of RNA data, thereby affecting the quality of different batches. This can lead to uncertain research conclusions being drawn from the experimental results. Therefore, it is necessary to use whole blood as a material to explore the influence of other processes on sequencing results.

As a supplement to the above study and to discover the mechanism behind these traits, our study aims to explore whether different storage times and processing methods for the same sample affect transcriptional expression results, and to thus provide a sample processing method recommendation for researchers.

## 2. Materials and Methods

### 2.1. Sample Collection and Processing

All samples were taken from healthy subjects at the School Hospital of Southeast University, who were aged between 20 and 30. All subjects reviewed and signed an informed consent form.

PBMCs from one individual (500 μL) were extracted using a Ficoll-Hypaque gradient after 0, 1, 4 and 8 h, at the usual centrifugation speed (1500× *g*) at room temperature. The samples included a male and two donors from the GEO database.

The blood samples were collected in accordance with regulations. Blood samples were collected in a K2-EDTA anticoagulant tube and incubated in a 4 °C constant-temperature refrigerator for 0, 4, 8, 24 or 32 h. The samples were from three males and three females.

All details about the samples are shown in Table 1.

### 2.2. Ribonucleic Acid (RNA) Isolation and Complementary Deoxyribonucleic Acid (cDNA) Library Construction

Total RNA from PBMCs (including samples from the GEO database) was extracted using TRIzol reagent (Thermo Fisher Scientific, Waltham, MA, USA), and blood RNA was extracted using the AM1928 RiboPure-Blood kit (Thermo Fisher Scientific, Waltham, MA, USA). RNA integrity numbers (RINs) were measured using the Agilent 2100 (Agilent Technologies, Santa Clara, CA, USA). The quality was classified from one to 10, where one represents the most serious degradation and 10 represents no degradation. Different degrees of RNA degradation would affect the transcriptome results; the high-quality standard for RNA sequencing requires the minimum value to be eight [10,17,18].

The cDNA library-construction method was consistent with our previously published articles [19]. Firstly, 1 μg RNA was purified with 1.8 × RNA Clean Beads (Vazyme, China) to remove the genomic DNA and rRNAs. The cDNA library was constructed using a NEBNext^®^ UltraTM II Directional RNA Library Prep Kit for Illumina^®^ (New England Biolabs GmbH, Ipswich, MA, USA); the first cDNA strand and the second cDNA strand were synthesized, the end repair process and adapter ligation steps were undertaken, and finally, the polymerase chain reaction (PCR) enrichment of the adaptor-ligated DNA was undertaken. PCR products were purified using NEBNext Sample Purification Beads (New England Biolabs GmbH, Ipswich, MA, USA). Finally, the library was sequenced on an Illumina HiSeq X10 PE150 platform (Illumina, Inc. US Illumina, San Diego, CA, USA) with a paired-end pattern and insert sizes of 300 bp. The sequencing reads of all samples (including samples from the GEO database) are presented in Table 1. In addition, the sequencing read length of the samples from the GEO database was also 300 bp, which was consistent with the sequencing strategy in this study.

### 2.3. Bioinformatics Analysis

We used the fastp (v0.23.2) tool to perform sequencing quality control and filtering on the raw reads, resulting in clean reads. The clean reads were then aligned to the Hg19 reference genome using Hisat2(v2.1.0) with default parameters. Later, the reads number of every gene were counted using featureCounts (v2.0.1); the gene set we used was obtained from The Human GENCODE Gene Set and the version is consistent with the reference. The matrix of different gene counts in different samples was exported into an R package named edgeR (3.38.2). Zero-count genes in all of the samples were discarded, and each sample was divided into groups for comparison. Only those with log2 fold changes greater than 1 and adjusted *p*-values less than 0.05 were reserved.

We used RSEM (v1.3.1) tools to quantify gene expressions. RSEM uses the relationship between paired end, the length of reads, the length distribution of fragments and the quality value to establish the maximum likelihood abundance estimation model, based on the maximum expectation algorithm, in order to distinguish which transcripts are different subtypes of the same gene.

### 2.4. Analysis of the Similarities and Differences

We aimed to explore the impact of sample processing on blood RNA sequencing and the extent of the impact, and to explore the impact of commonly used bioinformatics analysis methods on the results. These variables are not completely uniform samples; they have been or are being used as standard samples for scientific research, and these conventional analysis methods are used to obtain seemingly credible results.

Based on these methods, similarity and difference analyses and regular extraction should be carried out on RNA sequencing results obtained using different sample processing methods but from the same individual, so as to clarify whether sample processing methods have an impact on sequencing results and, if so, the extent of their impact.

#### 2.4.1. Correlation Analysis

The correlation of gene expression level among samples is an important index to test the reliability of the experiment and the rationality of sample selection. The closer the correlation coefficient is to one, the higher the similarity is. According to the quantitative results of FPKM, we calculated the correlation between the two samples, and made a heat map of the correlation. The degree of similarity is indicated by the color depth. The deeper the red color is, the greater the difference between the two samples is. The lighter the red color is, the more similar it is.

#### 2.4.2. Gene Clustering

We used the Euclidean distance algorithm to calculate the expression distance of each sample gene, and used the dispersion squared (Ward) algorithm to calculate the distance between samples, using the default parameters of hierarchical clustering from R studio. We established a clustering map, according to distance, that can intuitively reflect the distance relationship between the samples to show the difference between them.

#### 2.4.3. Differential Gene Screening

Referring to the sequencing-based differential gene detection method published by Audic S. et al. [20], in our analysis, the default definition of a differential expression gene is FDR ≤ 0.001, and multiple difference is defined as more than two times, based on a Poisson distribution.

#### 2.4.4. Time-Sensitive Genes

In order to find time-sensitive genes, we used the edgeR package (3.38.2) to find the intersections of two different genes between samples 0, 4 and 8, with three different storage time periods. Then, five genes with the most significant changes over time were selected. In order to further verify the accuracy of the results, we performed the same operation for other GEO sequencing data and focused on whether these five genes met the expected trend.

#### 2.4.5. Gene Enrichment Analysis

Based on the genome annotation information, genes with the same or similar functions are enriched to reduce their dimensionality. We used the clusterProfiler package (4.4.1) to perform GO and KEGG analyses on the selected differential genes; please refer to official documents for the detailed operations.

## 3. Results

### 3.1. Gene Expression Changes along with Storage Times

**Figure 1:**
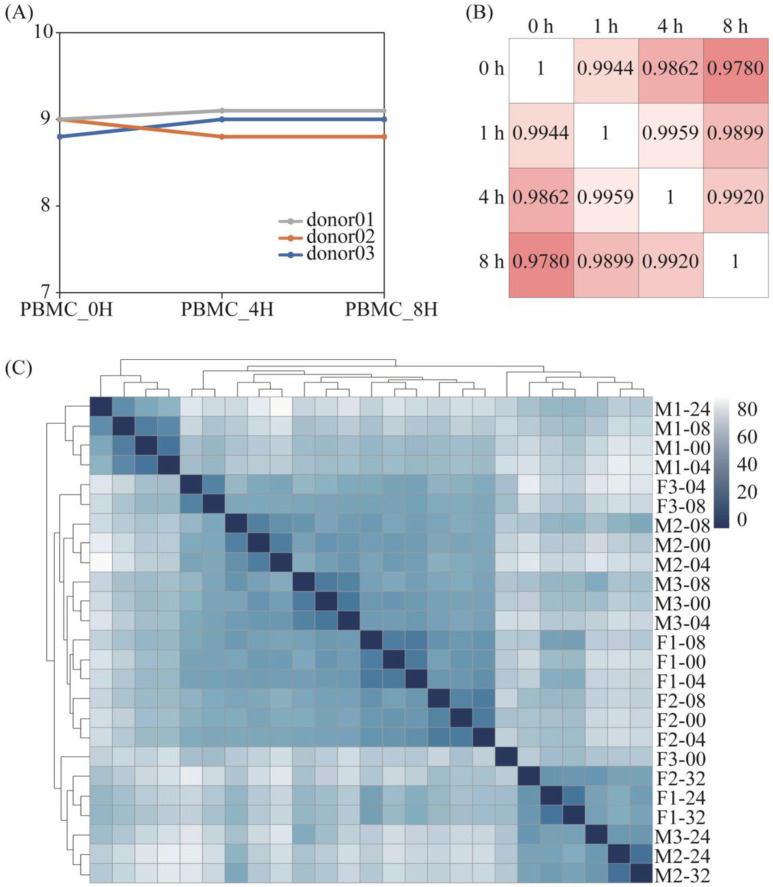
RNA integrity and correlation of differential treated samples. (**A**) The RNA integrity number extracted from PBMCs samples. The gray line represented donor 01. The blue and orange lines represented donor 02 and donor 03. (**B**) Correlation coefficient diagram of PBMCs samples from donor 01. The intensity of the color represented the similarity. (**C**) Gene clustering of RNA-Seq data from whole blood samples (F means female, M means male, 1–3 represent the numbers of different donors).

The RNA integrity numbers (RINs) among PBMC samples did not differ significantly. All of them were between 8.5 and 9.5 (Figure 1A), which meant the RNA quality was sufficiently maintained. By comparing the transcriptome gene differences for different treatments, we found that the gene expression difference was higher for the same genes with an increased storage time, and this difference was most obvious at the 8-h point, while there was no significant change in the total number of RNAs within 8 h.

The correlation analysis (Figure 1B) showed that the correlation between the total genes within 8 h was greater than 0.97, and that the minimum was 0.978 within 8 h. When comparing the overall sequencing differences in PBMCs that were extracted from blood samples stored within 8 h, the results of the above correlation analysis showed that there were no significant differences at four distinct time points. However, Dvinge concluded that sample processing caused widespread genomic alterations [14]; nonetheless, this is not contradictory because, while the overall results are similar, this does not necessarily mean that the gene expression results of the samples are the same.

For the whole blood samples, it can be seen from the sample cluster graph that, within the first 8 h, the difference between different individuals is greater than the difference in storage time. At the same time, even if some individuals exceed 8 h, the basic expression gap is greater than the storage time. In general, the difference in gene expression within 8 h of storage time is mainly due to individual differences, and, after 8 h, it is mainly due to storage time. There was no significant change among the samples with the increase in storage time (Figure 1C).

Briefly, the gene expression differences gradually rose with the increasing storage time, while there was no significant change in the total number of RNAs within 8 h.

### 3.2. Five Genes Are Significantly Different within Eight Hours in Peripheral Blood Mononuclear Cells (PBMCs)

**Figure 2:**
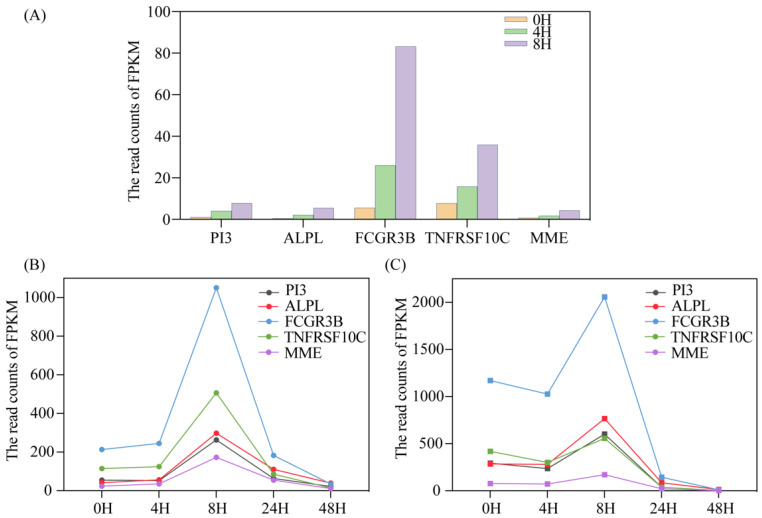
Five selected genes’ expression levels and their changes with different storage times. (**A**) The five genes showed significantly different FPKM within 8 h of PBMCs from donor 01; (**B**,**C**) The five genes showed significantly different FPKM within 24 h from donor 02 and donor 03.

To our surprise, there were still five genes with regular significant differences in gene expression following a short period of 8 h. All of them belonged to the genes encoding protein. These may be related to a series of stress responses after the cells are isolated in vitro. As shown in Figure 2A below, the expression levels of five genes gradually increased over time. These five genes were as follows: *peptidase inhibitor 3* (*PI3*), *alkaline phosphatase, bio-mineralization associated* (*ALPL*), *Fc fragment of the IgG receptor IIIbF* (*FCGR3B*), *TNF receptor superfamily member 10c* (*TNFRSF10C*) and *membrane metalloendopeptidase* (*MME*).

The *PI3* gene encodes an elastase-specific inhibitor as an antimicrobial peptide against Gram-positive and Gram-negative bacteria and fungal pathogens. The protein contains four disulfide core (WFDC) domains of the WAP type, so it is a member of the WFDC domain family. Most of the WFDC gene members are located in the following two clusters on chromosome 20q12-q13: centromere and telomere. The gene belongs to the centromere cluster.

The *ALPL* gene encodes a protein of the alkaline phosphatase family, which plays a key role in bone mineralization by regulating the level of diphosphate (PPI). The product of this gene is a membrane-bound glycosylase, which is not expressed in any specific tissue. There are at least four different, but related, alkaline phosphatases, as follows: intestinal, placental, placental and liver/bone/kidney (tissue-nonspecific).

The protein encoded by the *FCGR3B* gene is a low-affinity receptor in the Fc region of γ immunoglobulin (IgG). As a monomer, the coding protein can bind to the monomeric or aggregated IgG. In contrast to FCGR3A, it cannot mediate antibody-dependent cytotoxicity and phagocytosis and may capture immune complexes in peripheral circulation.

The protein encoded by the *TNFRSF10C* gene is a member of the tumor necrosis factor receptor superfamily. This receptor cannot induce apoptosis, and is considered to be an antagonistic receptor, which can protect cells from TRAIL-induced apoptosis. This gene is expressed in many normal tissues, but not in most tumor cell lines, which may explain the specific sensitivity of tumor cells to TRAIL-induced apoptosis.

The protein encoded by the *MME* gene is a type II transmembrane glycoprotein and a common acute lymphocytic leukemia antigen. It is an important cell surface marker for the diagnosis of acute lymphocytic leukemia. The protein is a neutral endopeptidase, which can cleave peptides on the amino side of hydrophobic residues and inactivate several peptide hormones, including glucagon, enkephalin, substance P, neurotensin, oxytocin and bradykinin.

Among the above genes, three genes (*FCGR3B*, *TNFRSF10C* and *MME*) that changed greatly with the storage time were directly related to the immune and apoptosis pathways. This may indicate that the blood cells can still maintain a long period of activity under the protection of anticoagulants after blood isolation. However, under the stimulated in vitro environment, they may induce some self-protection functions and a sacrificial apoptosis mechanism of the PBMCs, so as to ensure the maintenance of the basic vitality of cells. At the same time, this result suggests that the expression of some genes changes significantly with storage time. If we do not control the time and method of sample acquisition, it may lead to inconsistent sequencing results, or even interfere with the accuracy of disease feature signal molecules acquisition.

The important metabolic pathways and enrichment analysis of these five genes are briefly described in Table 2, the information in which was taken from the website ‘Gene cards’.

We analyzed the GEO sequencing (Accession nos. GSE58335) data from the same individuals but under different storage times, based on the same bioinformatics protocols, and found that the trend of FPKM over time was consistent with the above results (Figure 2B,C). When the storage time lasts approximately 8 h, there will be a peak in gene expression; and, as the storage time continues, the read counts will gradually decrease to close to 0. This suggests that PBMCs may have a stress-enhancing response for a period of time in vitro, and that some specific genes may be inactivated after they play a role. This is also consistent with the results we found earlier; these five genes are mainly related to immune and apoptotic pathways. After blood separation, blood cells can still maintain long-term activity under the protection of anticoagulants. However, under the stimulation of the external environment, some self-protection functions of PBMCs can be induced, sacrificing the mechanism of cell apoptosis, and thereby ensuring the maintenance of basic cell vitality; but, over time, these genes gradually become inactive.

### 3.3. Sequencing Differences between Whole Blood and Peripheral Blood Mononuclear Cells (PBMCs)

**Figure 3:**
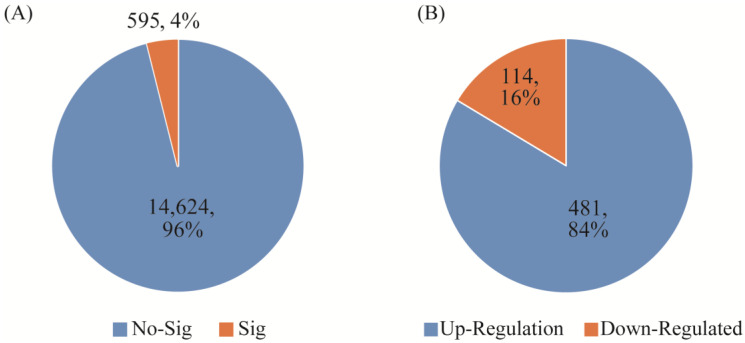
Comparison of the difference between whole blood and PBMCs sample extraction. (**A**) the number of significantly different genes between whole blood and PBMCs. (**B**) the number of up-regulated and down-regulated genes between whole blood and PBMCs.

Using the same bioinformatics processing method for different samples and using the edgeR package (3.38.2) to analyze whole blood and PBMC sequencing samples, the *p* value was set to 0.05, and 595 differential genes were found. Among them, 114 genes were upregulated and 481 genes were downregulated. There were no significant differences between 14,624 genes. As shown in Figure 3 below, it is not difficult to see that even samples from the same individual, using different sample processing methods, still have large differences; these results are likely to affect the conventional differential gene expression screening and lead to erroneous results.

**Figure 4:**
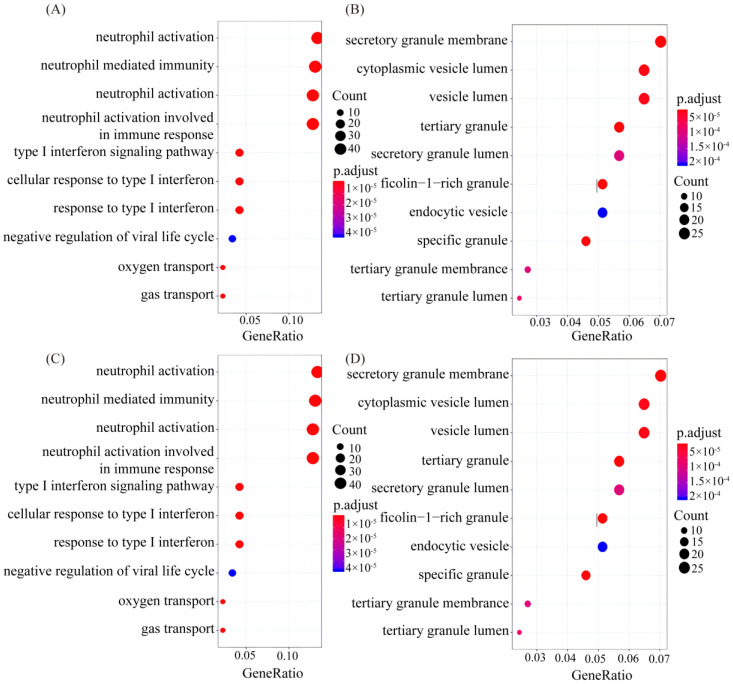
Differential expressed gene of Go and KEGG diagrams between whole blood and PBMCs samples. (**A**–**C**) denoted differential gene GO analysis of biological processes, cell components and molecular functions of the top 10 genes, respectively, *p* < 0.05; (**D**) key genes in KEGG pathway.

Then, GO and KEGG analyses were performed on these differential genes (Figure 4). The gene function annotation analysis showed that, in the biological process, neutrophils activate, mediate and participate in immune response and degranulation the most, and the type I interferon and its signaling pathways and cellular reactions are second. These are followed by the negative regulation of the virus cycle, oxygen transport and gas transport. The different gene functions of the cellular component mainly focus on the secretion of granular membranes, cyst cavity and cytoplasm cyst cavity, with other secretion-related components. The differences in the composition of molecular functions mainly focus on the binding of immunoglobulins to chemokines and transferase activity. The KEGG pathway analysis showed that differential genes were enriched in metabolic pathways related to phagosomes, malaria, viruses, cytokines and receptors, and Leishmaniasis.

## 4. Discussion

In previous studies, as long as the same person’s sample was used as a biological experiment and as a control sample, the influence of time and treatment method was rarely considered. In the above experiments, PBMCs were used to extract blood samples from the same person after different storage times. The results showed that the preservation of the samples within 8 h had little effect on most gene expression screening.

However, there are still some genes sensitive to preservation time that need special attention. We screened five time-sensitive genes and found that they are not only related to leukemia, immune neutropenia and other hematological processes, but also related to immune diseases and inflammatory reactions. This suggests that we should not only consider the changes in disease type and normal type, but also consider the change in preservation itself. Therefore, the research of related disease and cancer needs strict control time.

The same blood can use different extraction methods to obtain very different results. If the standard experimental procedure does not pay attention to the unification of sample pre-processing, it is likely to obtain deviating or misleading results. Since whole blood has a lot more hemoglobin than PBMCs, its differential genes may be related to hemoglobin production and oxygen transport. Neutrophils, chemokines, disease and virus-related genes and metabolic pathways may be related to the stress of PBMCs in vitro.

Our research suggests that sample preservation and processing in scientific research should not be an evasive issue, especially when looking for differential molecules and cancer treatment targets. This also means that the source of the sample should be as strict as possible. Sometimes, the overall difference is not large, but individual details may cause errors in the results.

This study has summarized a set of blood sample processing strategies to reduce the impact of sample processing on the results of RNA sequencing. The strategies are summarized as follows:

1. Try to select individuals with similar age ranges, genders and health statuses as blood sequencing samples. It is necessary to classify and discuss them when conducting research;
2. After the blood sample is collected, try to extract RNA as soon as possible. If the experiment cannot be carried out immediately, it can be stored in an ice box at about 4 °C and added later with stabilizer RNA and anticoagulant;
3. When studying genes or diseases related to leukemia, immune neutropenia and other blood and immune diseases and inflammatory reactions, the consistency of storage time should be strictly controlled;
4. When extracting RNA from whole blood, try to select a reagent kit that can remove hemoglobin as much as possible.

## Figures and Tables

**Table 1:** Samples from healthy donors.

Donor	Sex	Sample Type	Treatment	Hours	Million Reads	RIN
01	XY	PBMCs	room temperature	0	23.9	8.8
				1	24.0	9.1
				4	24.0	9
				8	23.9	9
02 ^1^	XX	PBMCs	room temperature	0	35.4	9.0
				4	33.0	8.8
				8	36.9	8.8
03 ^1^	XY	PBMCs	room temperature	0	48.2	9.0
				4	45.1	9.1
				8	46.3	9.1
04	XX	blood	4 °C	0	45.4	8.9
				4	48.0	9.0
				8	49.0	8.7
				24	48.2	8.0
05	XX	blood	4 °C	0	48.7	8.8
				4	47.0	8.9
				8	48.7	8.6
				24	47.0	8.3
06	XX	blood	4 °C	0	48.1	9.0
				4	46.1	8.7
				8	47.4	8.7
				24	47.1	8.5
				32	45.9	7.9
07	XY	blood	4 °C	0	45.5	8.9
				4	47.2	8.7
				8	47.0	8.5
				24	47.3	8.5
				32	46.0	8.0
08	XY	blood	4 °C	0	46.1	9.2
				4	48.7	9.1
				8	48.6	8.7
				32	46.8	7.8
09	XY	blood	4 °C	0	45.7	9.1
				4	47.5	9.0
				8	45.9	8.6

^1^ Donors from the GEO database.

**Table 2:** Metabolic characteristics and diseases related to five genes.

Name	Description	Diseases	Pathways	GO Annotations
*PI3*	Peptidase Inhibitor 3	Adult Respiratory Distress Syndrome; Bacterial Infectious Disease; Cystic Fibrosis; Inflammatory Bowel Disease; Joubert Syndrome 7; Lung Disease; Pasteurellosis; Preterm Premature Rupture of The Membranes; Skin Disease	Keratinization; Innate Immune System; Defensins; Nervous system development	endopeptidase inhibitor activity; serine-type endopeptidase inhibitor activity; structural constituent of skin epidermis; peptidase inhibitor activity
*ALPL*	Alkaline Phosphatase, Biomineralization Associated	Hypophosphatasia, Childhood; Hypophosphatasia, Infantile; Hypophosphatasia, Adult; Prenatal Benign Hypophosphatasia; Primary Bone Dysplasia	Endochondral ossification with skeletal dysplasias; Metabolism of proteins; Phenytoin Pathway, Pharmacokinetics; NAD metabolism; Post-translational modification: synthesis of GPI-anchored proteins; FGF23 signaling in hypophosphatemic rickets and related disorders; Netrin-UNC5B signaling pathway; NOTCH1 regulation of endothelial cell calcification; OSX and miRNAs in tooth development	alkaline phosphatase activity; inorganic diphosphate phosphatase activity; calcium ion binding; protein binding; pyrophosphatase activity
*FCGR3B*	Fc Fragment of IgG Receptor IIIb	Paroxysmal Nocturnal Hemoglobinuria; Neutropenia; Cryptococcosis; Poliomyelitis; Hemoglobinuria; Hypersensitivity Vasculitis; Peritonitis	Innate Immune System; Metabolism of proteins; GPCR Pathway; Fc-GammaR Pathway; RhoGDI Pathway; Integrin family cell surface interactions; Post-translational modification: synthesis of GPI-anchored proteins	transmembrane signaling receptor activity; IgG receptor activity; IgG binding; GPI anchor binding
*TNFRSF10C*	TNF Receptor Superfamily Member 10c	46, Xy Sex Reversal 8; Ovarian Cancer; Myelodysplastic Syndrome; Prostate Cancer; Myeloma, Multiple	Gene expression (Transcription); Akt Signaling; TGF-β Pathway; MIF Mediated Glucocorticoid Regulation; TNFR1 Pathway; TP53 Regulates Transcription of Cell Death Genes; PAK Pathway; ERK Signaling; Death Receptor Signaling Pathway; Regulation by c-FLIP	transmembrane signaling receptor activity; protein binding; TRAIL binding
*MME*	Membrane Metalloendopeptidase	Spinocerebellar Ataxia 43; Charcot-Marie-Tooth Disease, Axonal, Type 2t; Charcot-Marie-Tooth Disease, Axonal, Type 2e; Membranous Nephropathy; Peripheral Nervous System Disease	Innate Immune System; Cardiac conduction; Peptide hormone metabolism; Alzheimer’s disease and miRNA effects; ACE Inhibitor Pathway, Pharmacodynamics; Metabolism of proteins; A-β Plaque Formation and APP Metabolism;	peptidase activity metallopeptidase activity; protein binding; cardiolipin binding

## Data Availability

The original contributions presented in the study are included in the article, further inquiries can be directed to the corresponding author.
